# P-1641. Comparing Traditional and Weighted Incidence Combined Syndromic Antibiograms in Nursing Homes

**DOI:** 10.1093/ofid/ofae631.1807

**Published:** 2025-01-29

**Authors:** Lindsay Taylor, Sally Jolles, Taissa A Bej, Corinne Kowal, Oteshia Hicks, Ronald Gangnon, Jon P Furuno, David A Nace, Robin Jump, Christopher J Crnich

**Affiliations:** University of Wisconsin School of Medicine and Public Health, Madison, Wisconsin; University of Wisconsin School of Medicine and Public Health, Madison, Wisconsin; Louis Stokes Cleveland VA Medical Center, Cleveland, Ohio; Louis Stokes Cleveland VA Medical Center, Cleveland, Ohio; VA Northeast Ohio Healthcare System, Cleveland, Ohio; University of Wisconsin-Madison, Madison, Wisconsin; Oregon State University, Portland, Oregon; University of Pittsburgh, Pittsburgh, Pennsylvania; VA Northeast Ohio Healthcare System, Cleveland, Ohio; University of Wisconsin School of Medicine and Public Health, Madison, Wisconsin

## Abstract

**Background:**

Aggregate susceptibility reports, commonly referred to as antibiograms, may assist empiric antibiotic decisions. Creating traditional antibiograms (TAs) is hampered by the limited number of cultures collected in NHs. A weighted incidence syndromic combined antibiogram (WISCA) is an alternative method of representing susceptibility data. We compared TAs and WISCAs in a sample of NHs from three U.S. regions.

Table: Success Rate of Creating a TA and WISCA Using One Year of Culture Data in 75 Nursing Homes

**Figure:**
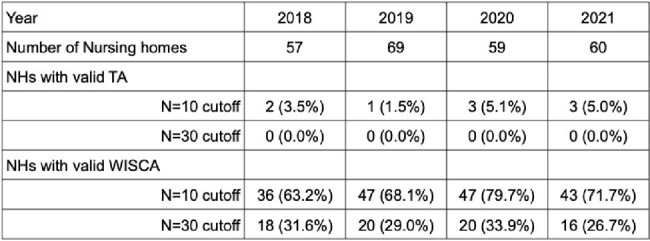

**Methods:**

Data on urine cultures collected in 75 NHs in Ohio, Oregon, and Pennsylvania (2018-2021) were obtained from their reference laboratories. Clinical & Laboratory Standards Institute (CLSI) breakpoints were used to normalize susceptibility interpretations across laboratories. We attempted to create a TA and WISCA for each study NH using the 3 most prevalent uropathogens (*Escherichia coli, Proteus mirabilis,* and *Klebsiella pneumoniae*) and susceptibilities to 5 commonly prescribed antibiotics. Sensitivity analyses using different isolate number thresholds (10 & 30) and years of culture data (1 & 2) were conducted.

**Figure:**
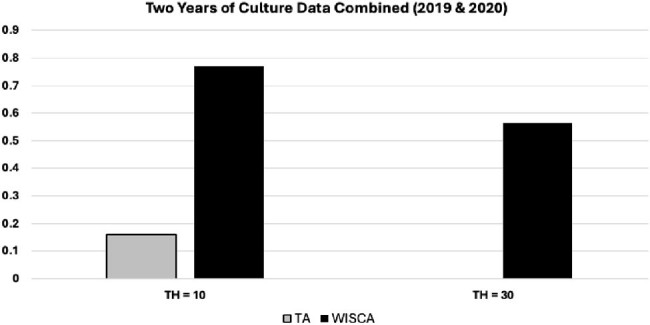
Success Rate of Creating a TA and WISCA Using Two Years of Culture Data in 75 Nursing Homes

**Results:**

15,238 unique urine cultures obtained from 7,703 residents were obtained from reference laboratories. The final sample was 12,789 cultures after excluding duplicate isolates obtained from the same resident within 3 monthsof an index urine culture. A TA could be constructed for ∼5% (1.5% - 5.1%) of NHs using a single year of culture data and an isolate number threshold of 10 (**Table**). This increased to ∼11% when combining two years of culture data (**Figure**). A 1- or 2-year TA could not be constructed for any of the NHs using the CLSI recommended isolate threshold of 30. We were able to construct a WISCA for most of the study NHs (63% - 80%) using a 10-isolate threshold and this improved slightlyby combining two years of culture data. We were only able to construct a 1-year WISCA for a third of NHs (29% - 34%) using a 30-isolate threshold but this improved to 57% of NHs when combining 2 years of culture data.

**Conclusion:**

A facility-specific urinary antibiogram can be produced for the majority of NHs by using a WISCA approach and combining more than one year of culture data. Additional study to examine the effects of pooling facility culture results on WISCA success rates and effects on susceptibility estimates are needed.

**Disclosures:**

**Jon P. Furuno, PhD**, Merck & Co., Inc: Grant/Research Support **Robin Jump, MD, PhD**, Abacus: Grant/Research Support|Merck: Grant/Research Support|Pfizer: Advisor/Consultant|Pfizer: Grant/Research Support

